# Structural and Functional Analyses of PAS Domain Interactions of the Clock Proteins *Drosophila* PERIOD and Mouse PERIOD2

**DOI:** 10.1371/journal.pbio.1000094

**Published:** 2009-04-28

**Authors:** Sven Hennig, Holger M Strauss, Katja Vanselow, Özkan Yildiz, Sabrina Schulze, Julia Arens, Achim Kramer, Eva Wolf

**Affiliations:** 1 Max Planck Institute of Molecular Physiology, Department of Structural Biology, Dortmund, Germany; 2 Max Planck Institute for Colloids and Interfaces, Potsdam, Germany; 3 Laboratory of Chronobiology, Charité - Universitätsmedizin Berlin, Berlin, Germany; Vanderbilt University, United States of America

## Abstract

PERIOD proteins are central components of the *Drosophila* and mammalian circadian clocks. The crystal structure of a *Drosophila* PERIOD (dPER) fragment comprising two PER-ARNT-SIM (PAS) domains (PAS-A and PAS-B) and two additional C-terminal α-helices (αE and αF) has revealed a homodimer mediated by intermolecular interactions of PAS-A with tryptophane 482 in PAS-B and helix αF. Here we present the crystal structure of a monomeric PAS domain fragment of dPER lacking the αF helix. Moreover, we have solved the crystal structure of a PAS domain fragment of the mouse PERIOD homologue mPER2. The mPER2 structure shows a different dimer interface than dPER, which is stabilized by interactions of the PAS-B β-sheet surface including tryptophane 419 (equivalent to Trp482_dPER_). We have validated and quantitatively analysed the homodimer interactions of dPER and mPER2 by site-directed mutagenesis using analytical gel filtration, analytical ultracentrifugation, and co-immunoprecipitation experiments. Furthermore we show, by yeast-two-hybrid experiments, that the PAS-B β-sheet surface of dPER mediates interactions with TIMELESS (dTIM). Our study reveals quantitative and qualitative differences between the homodimeric PAS domain interactions of dPER and its mammalian homologue mPER2. In addition, we identify the PAS-B β-sheet surface as a versatile interaction site mediating mPER2 homodimerization in the mammalian system and dPER-dTIM heterodimer formation in the *Drosophila* system.

## Author Summary

Most organisms have daily activity cycles (circadian rhythms), which are generated by circadian clocks. Circadian periodicity is produced by specific clock protein interactions and posttranslational modifications as well as changes in their cellular localization, expression, and stability. To learn more about the molecular processes underlying circadian clock operation in fruit flies and mouse, we analysed the homo- and heterodimeric interactions of the clock proteins *Drosophila* PERIOD (dPER) and mouse PERIOD2 (mPER2). We show that dPER and mPER2 use different interaction surfaces for homodimer formation, which are associated with different dimerization affinities. In addition, we present a structure-based biochemical analysis of the heterodimeric interaction of dPER with its partner *Drosophila* TIMELESS (dTIM). We identify a versatile molecular surface of the PERIOD proteins, which mediates homodimer formation of mPER2 but is used for dPER-dTIM heterodimer formation in *Drosophila*. Our results reveal quantitative and qualitative differences in the molecular interactions of PERIOD clock proteins in flies and mammals, allowing them to adjust to their different binding partners and regulatory functions in these different organisms.

## Abstract 3

Crystal structures and structure-based biochemical studies of*Drosophila* PERIOD and mouse PERIOD2 circadian clock proteins reveal different homodimer interactions and identify a versatile molecular surface that mediates homodimerization of mouse PERIOD2 but is involved in heterodimeric interactions of*Drosophila* PERIOD with TIMELESS.

## Introduction

**Figure 1:**
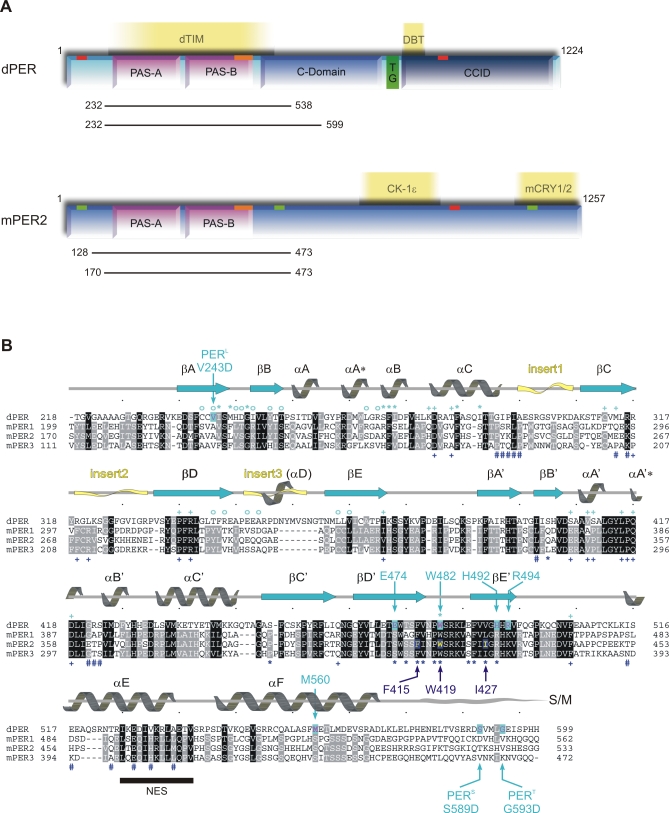
Domain Architecture and Sequence Alignment of dPER and Mouse PERIOD Proteins (A) Domain architecture of dPER and mouse PERIOD2 (mPER2). The two PAS domains (PAS-A and PAS-B); the cytoplasmic localization domain (CLD, orange bar); nuclear localization signals (NLS, red bars); NES (green bars); the conserved C-domain; the threonine-glycine (TG) repeat region; and the dCLK:CYC inhibition domain (CCID) of dPER and/or mPER2 are shown schematically. CKIɛ, mCRY1/2, dTIM, and DBT are depicted at their binding sites. The dPER and mPER2 fragments used for crystallization and biochemical studies are represented as black bars. (B) Sequence alignment of the PAS-A and PAS-B domains as well as helices αE and αF of dPER (Swissprot accession number P07663) with mPER 1, 2, and 3 (Swissprot accession number [1] O35973, [2] O54943, and [3] O70361). Secondary structure elements were assigned from the dPER[232–599] structure ([31], PAS-A, βA → βE; PAS-B, βA′→ βE′). Partially disordered insert regions are depicted as yellow waves. The sequence alignment was generated in ClustalW [78]. dPER residues Trp482, Met560, Glu474, His492, Arg494 mutated herein as well as the sites of the *per* ^L^ long period mutation V243D and the *per* ^S/T^ short period mutations S589D and G593D are highlighted by cyan arrows, mutated mPER2 residues Trp419, Ile427, and Phe415 by dark blue arrows. dPER residues in the Trp482 binding pocket of PAS-A are marked with cyan stars (*), dPER residues interacting with the αF helix with cyan open circles (°). Residues in the dimer interfaces of mPER2 are marked with dark blue stars (*, PAS-B β-sheet interface) or hashes (#, PAS-A dimer interactions). Residues involved in intramolecular PAS domain interactions are marked with cyan (dPER) and dark blue (mPER2) plus signs (+). S/M, short-mutable region.

In adaptation to daily environmental changes most organisms ranging from cyanobacteria to humans display 24-h day-night activity cycles, so called circadian rhythms. In humans, many physiological and behavioural processes such as the sleep-wake cycle, body temperature, blood pressure, hormone production, and the immune system are regulated in a circadian manner. Circadian rhythms are generated by circadian clocks (also referred to as circadian oscillators), which are operated by interconnected transcriptional-translational feedback loops [1–3]. A structural feature shared by many eukaryotic clock proteins are the PER-ARNT-SIM (PAS) domains [4,5], which represent a diverse and ubiquitous family of sensory-, signalling-, and protein-protein interaction modules. Examples include the *Drosophila* (d) and mouse (m) PERIOD proteins (Figure 1A) as well as the bHLH-PAS transcription factors d/mCLOCK, dCYCLE, and mBMAL1, which contain two tandemly organized PAS domains (referred to as PAS-A and PAS-B) for protein-protein interactions. In the circadian oscillator of D. melanogaster, expression of the clock protein PERIOD (dPER) [6] and its interaction partner TIMELESS (dTIM) is activated by the heterodimeric bHLH-PAS transcription factors dCLOCK and dCYCLE. dPER and dTIM act as negative elements in the feedback loop by repressing their own transcription. Day-time dependent changes in concentration, cellular localization, and transcriptional repressor activity of dPER are essential for maintaining circadian rhythmicity. These changes are critically influenced by Doubletime (DBT)- [7–9] and casein kinase II (CKII)- [10,11] mediated phosphorylation of dPER as well as the interaction of dPER with dTIM [12–15]. While DBT-dependent phosphorylation of dPER on Ser47 enhances its proteasomal degradation [16], dTIM binding [15] as well as dephosphorylation by the phosphatases PP2A and PP1 [17,18] increase dPER stability. dPER-dTIM interactions are additionally reported to promote nuclear entry of the complex due to specific interactions of dTIM with a cytosolic localization domain (CLD) of dPER, which is located in the PAS-B domain [12].

In the mammalian/mouse clock, three PERIOD homologues (mPER1, mPER2, and mPER3) and two CRYPTOCHROMES (mCRY1, mCRY2) act as negative elements of the transcriptional feedback loop, their expression being regulated by the bHLH-PAS transcription factors mCLOCK and mBMAL1. The stability and cellular localization of the mouse PERIOD proteins is regulated by casein kinase Iɛ (CKIɛ)-dependent phosphorylation [19] as well as interactions with mCRY1 and mCRY2 [20–24] or between the mPER homologues [23,25,26]. Whereas the PAS domains of dPER are involved in dTIM binding [12,27], the PAS domains of mPER1, 2, and 3 mediate homo- and heterodimerization of the three PERIOD homologues in the mammalian circadian clock [25,26,28]. The functional importance of the PAS domains in mPER2 is demonstrated by the fact that mice homozygous for a mutation, in which residues Ala348 to Val434 in the PAS-B domain of mPER2 are deleted (*mper2* ^m/m^), display a shorter circadian period followed by a loss of circadian rhythmicity in constant darkness [29]. Furthermore, *mper2* ^m/m^ mice carrying the PAS-B deletion mutation are cancer prone [30].

**Figure 2:**
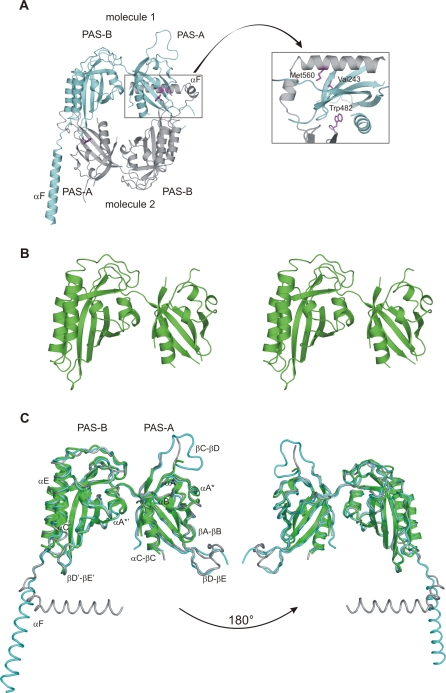
Crystal Structures of *Drosophila* PERIOD (A) Ribbon presentation of the dPER[232–599] dimer with molecule 1 shown in cyan, molecule 2 in grey. The inset shows a close-up view highlighting residues Trp482 and Met560, which have been mutated in this study. The *per* ^L^ mutation site (Val243) is also highlighted. (B) Stereo view of the dPERΔαF[232–538] monomer structure. (C) Superposition of the dPERΔαF[232–538] monomer structure (green) on molecule 1 (cyan) and 2 (grey) of the dPER[232–599] dimer. The two orientations are related by 180 ° rotations.

To provide insights into the PAS domain interactions of PERIOD proteins, we have previously solved the crystal structure of an N-terminal *Drosophila* PERIOD fragment, dPER[232–599] [31], including the two tandemly organized PAS domains (PAS-A and PAS-B) and two C-terminal α-helices, αE and αF, corresponding to residues 525–572 of the conserved C-domain of dPER (Figures 1 and 2A) [32]. The dPER[232–599] crystals contained a noncrystallographic dimer stabilized by interactions of the PAS-A domain with a conserved tryptophane residue, Trp482, in the βD′-βE′ loop of PAS-B (PAS-A–Trp482 interface) and with helix αF (PAS-A–αF interface). Interestingly, αF adopted different conformations in the two dPER[232–599] monomers, establishing intermolecular interactions to the PAS-A domain within the same dimer (αF of molecule 2) or to a symmetry-related dimer in the crystal (αF of molecule 1). In solution, dPER[232–599] behaves as a dimer, whereas a dPER construct lacking helix αF (dPERΔαF[232–538]) is monomeric [31]. In *Drosophila* flies, mutation of Val243 in the PAS-A domain to Asp (V243D, *per* ^L^) leads to a temperature dependent 29-h long-period phenotype [6]. Our dPER[232–599] structure revealed that Val243 is located in the centre of the PAS-A–αF interface packing with its side chain against Met560 of helix αF (Figure 2A). The *per^L^* mutation dissociated the dPER dimer in gel filtration analysis, presumably by introducing a negative charge (Asp243) into this hydrophobic interface [31]. Furthermore, the *per^L^* mutation and the mutation of Met560 to Asp lead to strong phenotypes in reporter gene assays and cellular localization studies conducted in Schneider 2 (S2)-cultured *Drosophila* cells [31]. These studies clearly demonstrated the existence of the PAS-A–αF dimer interface in solution and in full-length dPER within the cellular context. We therefore propose that the PAS-A–αF interaction plays a critical role in the circadian clock and that the 29-h phenotype of *per^L^* mutant flies is caused by a destabilization of this interface.

dPER homodimers had previously been observed in yeast-two-hybrid, co-immunoprecipitation (Co-IP), and crosslinking studies [32,33]. Moreover, small amounts of dPER homodimers were shown to be present in fly head extracts [14]. In the clock, homodimers might stabilize dPER in absence of dTIM and could potentially play a role in dTIM-independent transcriptional repression and cellular shuttling of dPER [34–38]. A detailed study of the functional role of the dPER homodimer in living flies is presented in the accompanying report by Landskron et al. [39].

In the mammalian/mouse clock, mPER1, 2, and 3 homo- and heterodimerize via their PAS domains [25,28], which display significant sequence-similarity to the PAS-A and -B domains of dPER (Figure 1B). Whereas Trp482 and residues mediating the intramolecular PAS domain interactions are well conserved in the mouse PERIOD proteins, the Trp482-interacting residues in the hydrophobic PAS-A binding pocket of dPER, the αF helix as well as the αF interacting residues of dPER are not (Figure 1B). It is therefore conceivable, that *Drosophila* and mammalian PERIOD proteins form structurally different homo- and heterodimers, allowing them to adjust to the different binding partners and regulatory processes in the *Drosophila* and mammalian clock systems.

To provide insights into the similarities and differences of the homo- and heterodimeric PAS domain interactions of dPER and mPER proteins in the *Drosophila* and mammalian circadian clock, we undertook a detailed structural and biochemical analysis of the PAS domain interactions of dPER and mPER2. We have determined the crystal structure of dPERΔαF, which (consistent with our gel filtration data) crystallizes as a monomer. Furthermore, we used site-directed mutagenesis, analytical gel filtration, and analytical ultracentrifugation to establish the significance of Trp482 for dimerization of dPER in solution and to quantify the relative contributions of the PAS-A–αF and PAS-A–Trp482 dimer interfaces. Using yeast-two-hybrid experiments, we show that dTIM interacts with the β-sheet surface of the PAS-B domain of dPER. To provide insights into the interactions of the PAS domains in the mammalian PERIOD proteins, we have determined the crystal structure of a fragment of mouse PERIOD2 (mPER2[170–473]), which contains the two PAS domains, the αE helix and a short piece N-terminal to the PAS-A domain. The structure shows a different noncrystallographic dimer than dPER, which is stabilized by interactions of the PAS-B β-sheet surface including Trp419 corresponding to Trp482_dPER_. Whereas the region equivalent to the αF helix of dPER is not required for mPER2 dimerization, our mutational analysis shows that the PAS-B/Trp419 dimer interface is important for mPER2 dimer formation in solution. Moreover we find a 100–200-fold higher affinity for the dimerization of mPER2 PAS domain fragments compared to dPERΔαF. Mutation of Trp419 to Glu dissociates the mPER2 PAS domain homodimer in HEK293 cells, suggesting that the PAS-B/Trp419 interface also mediates self association of mPER2 in a cellular context. Our study therefore identifies the PAS-B β-sheet surface as an interaction site used for both mPER2-mPER2 homodimerization and dPER-dTIM heterodimer formation.

## Results

### Crystal Structure of the dPERΔαF[232–538] Monomer

On the basis of our dPER[232–599] crystal structure, we have constructed and crystallized a shorter dPER fragment, dPERΔαF[232–538], containing the two PAS domains but lacking the αF helix. The crystal structure of dPERΔαF[232–538] was determined by molecular replacement using the refined dPER[232–599] structure as search model. Consistent with our gel filtration studies, dPERΔαF crystallises as a monomer. Overall, the structure of the dPERΔαF monomer is very similar to dPER[232–599] [31] with average root mean square deviations (rmsds) of 1.1 Å for 230 Cα positions (Figure 2B and 2C). In the dPERΔαF structure, the intramolecular PAS-A–PAS-B interactions are unchanged compared to the dimeric structure with helices αA′ and αA*′ of PAS-B packing against strands βC, βD, and βE and helix αC of PAS-A. Although traceable with confidence, helices αA, αA, and αB and the βA-βB loop forming the Trp482 binding pocket of PAS-A are represented by somewhat weaker electron density and higher B-factors, suggesting them to be more flexible in the monomeric structure. Furthermore, the βA-βB loop of PAS-A has changed its conformation compared to dPER[232–599], likely as a result of the missing dimer interaction with Trp482 of PAS-B. The circular dichroism (CD) spectra of dPERΔαF however closely resemble those of the dPER[232–599] fragment reflecting no changes of secondary structure composition apart from the missing αF helix (Figure S1). Consequently, dPER dimerization does not require major structural rearrangements in the two PAS domains, but stabilises the PAS-A fold by inserting Trp482 into its hydrophobic pocket and possibly also by covering the PAS-A β-sheet surface with the αF helix.

### Contribution of the PAS-A–αF- and PAS-A–Trp482 Interface to dPER Homodimer Formation

**Figure 3:**
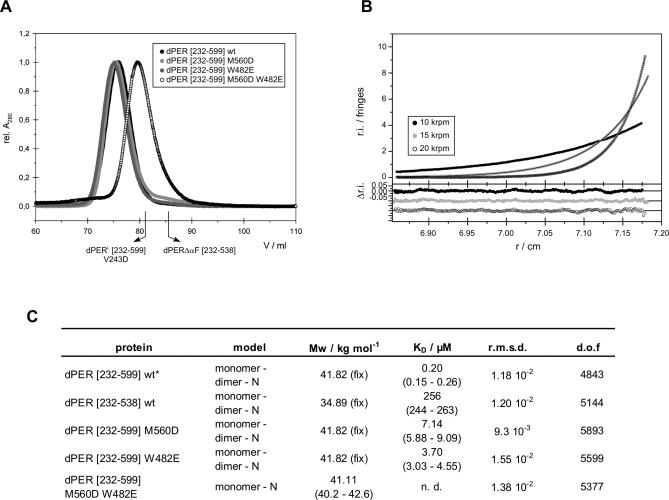
Analytical Gel Filtration and Ultracentrifugation of dPER (A) Analytical gel filtration of dPER[232–599] wild-type and mutants dPER(M560D), dPER(W482E), and dPER(M560D/W482E). Same amounts of *E. coli* expressed and purified proteins were loaded on a HiLoad Superdex 200 16/60 gel filtration column. The elution positions of the monomeric *dPER* ^L^[232–599]V243D mutant and dPERΔαF[232–538] [31] are indicated. (B) A typical sedimentation equilibrium experiment of dPER[232–599] wild-type at a single concentration (out of four). Data of other fragments and mutants were of comparable quality. (C) Summary table of analytical ultracentrifugation. *K*_D_ = 1/*K*_A_. dof, degrees of freedom; fix, molar mass fixed to the expected value. 95% confidence intervals are given in brackets. * A statistically equivalent description of the data is obtained for a single-ideal species model (rmsd of 1.12 × 10^−2^) but with a slightly lower molar mass (81.41 kg/mole, (81.22–82.13) kg/mol) than expected for the dimer (83.64 kg/mol).

In order to validate the importance of Trp482 for dPER dimerization in solution and to quantify the relative contributions of the PAS-A–αF and Trp482 interface to dPER dimerization, we have analyzed these interfaces by site-directed mutagenesis using analytical gel filtration and analytical ultracentrifugation (Figure 3). The mutants were designed to destabilize the PAS-A–αF interface (M560D), the PAS-A–Trp482 interface (W482E), or both of these hydrophobic interfaces (M560D/W482E) by introducing negative charges (Figure 2A). All mutations were introduced into the dPER[232–599] fragment. CD spectroscopic analysis of the purified wild-type and mutant PAS domain fragments revealed no changes in secondary structure content (Figure S1), suggesting that the point mutations did not affect the overall tertiary structure of the molecule.

Under gel filtration conditions (Figure 3A), the individual mutations M560D or W482E did not dissociate the dimer. In contrast, the double mutation M560D/W482E resulted in a monomeric species, indicating that both, the PAS-A–αF and the PAS-A–Trp482 interface are involved in dimerization in solution. Using analytical ultracentrifugation, we have determined the dissociation constants (*K*_D_ values) for dPER dimerization (Figure 3B and 3C). For the wild-type dPER[232–599] fragment, we measured a *K*_D_ of 0.2 μM. Removal of the complete αF helix in dPERΔαF[232–538] drastically changes the dissociation constant to 256 μM, confirming the importance of the PAS-A–αF interaction for dPER homodimerization. dPER[232–599] fragments carrying the single point mutations M560D and W482E have *K*_D_ values of 7.14 and 3.70 μM, respectively. This moderate reduction in affinity is consistent with their behaviour as dimers in gel filtration. In contrast to dPERΔαF, which still exhibits a monomer-dimer equilibrium, the M560D/W482E double mutant is completely monomeric. This likely reflects the fact that the Trp482 dimer interface is preserved in dPERΔαF but not in dPER[232–599]M560D/W482E.

### Crystal Structure of the mPER2 PAS Domains

**Figure 4:**
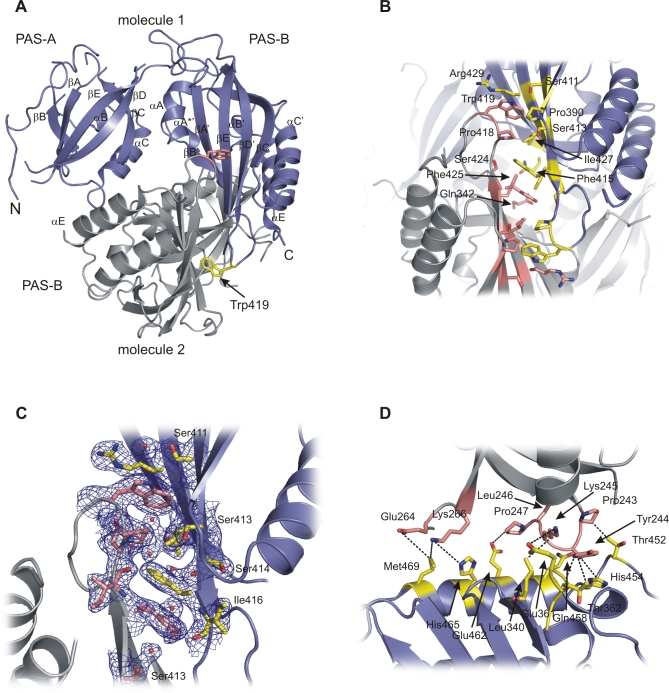
Structural Analysis of PAS Domain Interactions in mPER2[170–473] (A) Ribbon presentation of the mPER2[170–473] homodimer with molecule 1 shown in dark blue, molecule 2 in grey. The conserved Trp419 residues are shown as atomic stick figure. (B) Close-up view of the dimer interface formed by antiparallel packing of the PAS-B β-sheet surfaces. Interacting residues are highlighted as atomic stick figure. Residues Trp419, Ile427, and Phe415 have been mutated within this study. Top, molecule 1 in dark blue; bottom, molecule 2 in grey. (C) Close-up view of the dimer interface formed by antiparallel packing of the PAS-B β-sheet surfaces. Residues and water molecules mediating dimer interactions are highlighted as atomic stick figures and red spheres. Pro390, Phe425, and Glu342 have been omitted for clarity. Ser414 and Ile416 establish main chain interactions to water molecules in the interface. The 1 sigma 2fo-fc composite omit map is shown in blue. Similar view as in Figure 4B, but with molecules 1 and 2 switched/rotated around the noncrystallographic symmetry (NCS) axis. Top, molecule 2 in dark blue; bottom, molecule 1 in grey. (D) Close-up view showing dimer interactions of the PAS-A domain with the PAS-B domain and helix αE. Interacting residues are highlighted as atomic stick figure. Pairs of interacting residues (hydrogen bond or van der Waals interactions) are connected by dashed lines.

To provide insights into the PAS domain interactions of PERIOD proteins in the mammalian circadian clock, we have determined the crystal structure of the mouse PERIOD2 fragment mPER2[170–473], which includes the two PAS domains (PAS-A and PAS-B), the αE helix, and a short extension N-terminal to the PAS-A domain. The mPER2[170–473] structure was solved by molecular replacement using the refined dPER[232–599] structure as search model. The structure revealed a noncrystallographic dimer, which, in contrast to dPER, is mainly stabilized by interactions between the PAS-B β-sheet surfaces that pack against each other in an antiparallel orientation (Figures 4A and S2). Interestingly, the PAS-B dimer interface includes the conserved Trp419 (corresponding to Trp482 of dPER) of both monomers as a crucial interface residue (Figure 4A and 4B). In the homodimer, both Trp419 residues are 80% buried because of stacking interactions with the preceding Pro418 as well as van der Waals interactions with Ile427, Arg429, Pro390, and Ser413 of the dimerising molecule. Pro418 itself also packs against Ile427, Pro390, and Ser413 of the dimerising molecule. The side chain OH-group of Ser411 in molecule 1 forms a hydrogen bond to the side chain nitrogen of Trp419 in molecule 2 (Figures 4B and S3A, left panel), whereas Ser411 of molecule 2 coordinates one of the water molecules located in the mPER2 dimer interface (Figures 4C and S3A, right panel). Overall, the PAS-B dimer interface is predominantly hydrophobic and also includes two centrally located phenylalanines, Phe415 and Phe425, which establish stacking interactions to their noncrystallographic symmetry mates (Figures 4B and S3B). Nearby Phe425, the two Gln342 residues of molecules 1 and 2 form an H-bond interaction between their side chain carbonyl and amino groups.

In addition to the PAS-B β-sheet interactions, the mPER2 homodimer is stabilized by interactions of the PAS-A domain with helix αE and the PAS-B domain. Residues Pro243-Pro247 located C-terminal to the αC helix of PAS-A interact with the N-terminal end of the αE helix (Thr452, His454, Gln458, Glu462) as well as Leu340 in the βB′ strand and Glu361-Pro363 in the αA′-αB′ region of PAS-B (Figure 4D). Notably, the side chains of Tyr244 and Lys245 establish hydrogen bonds to the main chain carbonyl group and side chain carboxyl group of Glu361. Furthermore, His465 and Met469 in helix αE establish dimer interactions to residues Glu264 and Lys266 at the N-terminal end of the βC strand in PAS-A.

**Figure 5:**
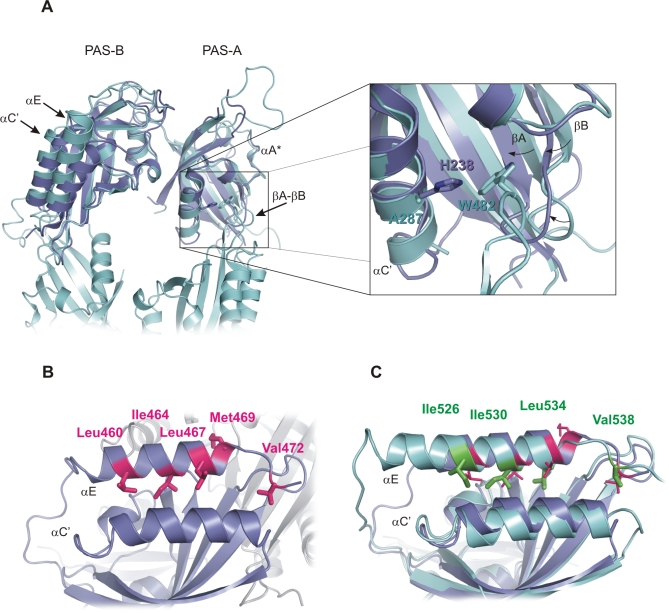
Structural Comparison of mPER2 and dPER Reveals a Putative NES in dPER (A) Left, superposition of molecule 1 of mPER2[170–473] (dark blue) and molecule 1 of dPER[232–599] (cyan, αF helix omitted). Largest changes are seen in the αE helix and the preceding linker, the αC′ helix as well as the hydrophobic pocket of PAS-A. Part of the dimerizing dPER[232–599] molecule 2 is shown in cyan. Right, superposition of the PAS-A–Trp482 dimer interface of dPER (cyan) with the PAS-A domain of mPER2 (dark blue), close-up view. His238 of mPER2 and Ala287 of dPER are shown as atomic stick figure. Trp482 of the dimerizing dPER molecule 2 is shown in cyan. Structural changes in strands βA and βB as well as the βA-βB loop are indicated by black arrows. (B) Close-up view of αE of mPER2 including an NES sequence. Residues Leu460, Ile464, Leu467, and Met469 of the NES sequence as well as Val472 (corresponding to Val538 of dPER) are shown as atomic stick figure. The dimerizing mPER2 molecule is shown in grey. (C) Superposition of the αE helix of mPER2 (dark blue) and dPER (cyan). The residues of the putative NES sequence in dPER (Ile526, Ile530, Leu534, Val538) are shown in green as atomic stick figure. Their location is compared to the NES residues Leu460, Ile464, Leu467, and Met469 as well as Val472 of mPER2 (magenta).

Each mPER2 monomer contains two canonical PAS domains with a five-stranded antiparallel β-sheet (βA-βE) covered on one face by α-helices (αA-αC). Except for some loop regions and the N-terminal extension, the two mPER2 monomers display no significant structural differences and can be superimposed with an rmsd of 0.76 Å for 241 Cα atoms (Figure S4). As expected based on the high conservation of residues involved in intramolecular PAS domain interactions (Figure 1B), the two PAS domains of mPER2 are arranged in a dPER-like manner. The mPER2 monomers can be superimposed on dPER with an rmsd of 1.66 Å (225 Cα positions) for molecule 1 (Figure 5A) and an rmsd of 1.71 Å (219 Cα positions) for molecule 2. Apart from disordered loop regions, the PAS-A domain of mPER2 is partially disordered in the region corresponding to the αA* helix in dPER. The hydrophobic pocket of PAS-A, which embeds Trp482 in dPER is somewhat more closed in mPER2. In addition to inward movements of the βA- and βB strands and the βA-βB loop, Ala287_dPER_ in the αC helix is replaced by a histidine (His238) in mPER2, changes that would likely sterically interfere with Trp419 binding (Figure 5A). Differences are also observed in the location of the αE helix and the preceding linker as well as in the αC′ helix. Notably, the αE helix of mPER2 contains a nuclear export signal (NES) comprising residues Leu460, Ile464, Leu467, and Met469 (Figures 5B and 1B) [40]. Whereas Leu460, Ile464, and Leu467 pack against the αC′ helix of PAS-B, Met469 points to the molecule surface and is involved in homodimer interactions (Figures 4D and 5B). The NES of mPER2 corresponds well to the NES consensus sequence L-x(2,3)-[LIVFM]-x(2,3)-L-x-[LI] and also includes a number of Glu, Asp, and Ser residues that are generally overrepresented in NES regions [41]. Sequence and structural comparison with dPER suggests that an equivalent NES function could be carried out by αE residues Ile526, Ile530, and Leu534 of dPER (corresponding to Leu460, Ile464, and Leu467 of mPER2), which also pack against the αC′ helix (Figure 5C). Met469_mPER2_ may be functionally replaced by Val538_dPER_ in the flexible loop connecting αE to αF. Interestingly, mPER2 also has a Valin (Val472) at the position equivalent to Val538_dPER_, whose functional role in nuclear export could be tested by site-directed mutagenesis.

In molecule 1, the PAS-A β-sheet surface is covered by an intramolecular interaction with residues 170–182 located N-terminal to the PAS-A domain (Figures 4A and S5). Although this N-terminal region does not adopt any particular secondary structure in mPER2, its interaction with the PAS-A domain likely replaces the dimer interaction of the αF helix in dPER (Figure S6, top left panel). Val176 and Glu177 of the N-terminal region cover and interact with Tyr204 of the PAS-A domain (Figure S5). Additionally, Tyr171 of the N-terminal region packs against Val294 and Trp249. In molecule 2, the N-terminal region as well as the side chains of Tyr204 and Trp249 are less ordered (Figure S4). Interestingly, Tyr253_dPER_ corresponding to Tyr204_mPER2_ stabilizes the kink of the αF helix in dPER. Moreover, Arg345_dPER_, which structurally overlaps with Val294_mPER2_, stabilizes the PAS-A–αF interaction in dPER by forming a salt bridge to Glu566_dPER_ in helix αF [31]. Therefore, residues Arg345_dPER_/Val294_mPER2_ and Tyr253_dPER_/Tyr204_mPER2_ appear to have nonconserved roles mediating intermolecular PAS-A–αF interactions in dPER, but intramolecular interactions between the PAS-A domain and N-terminal residues in mPER2.

### Analysis of mPER2 PAS Domain Interactions in Solution

**Figure 6:**
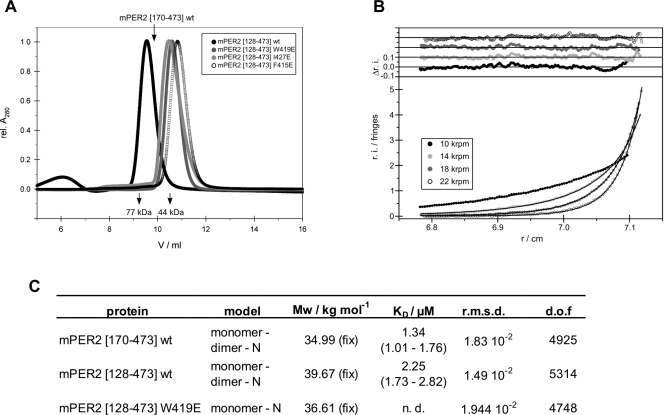
Analytical Gel Filtration and Ultracentrifugation of mPER2 (A) Analytical gel filtration of mPER2 interface mutants. Wild-type, W419E, I427E, and F415E mutant versions of mPER2[128–473] were analysed on a HiLoad Superdex 200 10/30 gel filtration column. The elution positions of the crystallized mPER2[170–473] fragment as well as the marker proteins Ovalbumin and Apotransferrin are indicated. (B) A typical sedimentation equilibrium experiment of dPER[128–473] wild-type at a single concentration (out of four). Data of other fragments and mutants were of comparable quality. (C) Summary table of analytical ultracentrifugation of mPER2 variants. *K*_D_ = 1/*K*_A_. dof, degrees of freedom; fix, molar mass fixed to the expected value. 95% confidence intervals are given in brackets.

To prove the existence of the mPER2 homodimer in solution and to determine the affinity for mPER2 homodimer formation, we have analysed the crystallized mPER2[170–473] fragment as well as an N-terminally extended PAS domain fragment of mPER2 (mPER2[128–473]) by analytical gel filtration and analytical ultracentrifugation. Interestingly both fragments behave as dimers in gel filtration analysis, despite the fact that they lack the region equivalent to the αF helix in dPER (Figure 6A). Moreover, their dissociation constants are 2.25 μM for mPER2[128–473] and 1.34 μM for mPER2[170–473], corresponding to a 110- and 187-fold higher affinity of homodimer formation compared to dPERΔαF (Figure 6B and 6C). To prove the importance of the PAS-B β-sheet surface for dimerization of mPER2 in solution, we have mutated residues Trp419, Ile427, and Phe415 located in the PAS-B interface (Figure 4B) to glutamate. In the homodimer, Trp419 and Pro418 establish van der Waals interactions to Ile427 in the PAS-B β-sheet surface of the dimerizing molecule. The W419E and I427E mutants are therefore expected to destabilize the dimer interface by removing the van der Waals interactions and introducing a negative charge in this hydrophobic interface region. The power of these mutations is further increased by the fact that, due to the 2-fold noncrystallographic symmetry, their contacts occur twice in the homodimer interface. The F415E mutation was designed to eliminate the stacking interaction between Phe415 and its noncrystallographic symmetry mate. Moreover, the negatively charged Glu415 side chain carboxyl groups of the two monomers would face each other in the homodimer and therefore most likely prevent its formation by electrostatic repulsion. Due to better yields of expression, the mutations were introduced into the N-terminally extended mPER2[128–473] fragment. (Figure 1A). CD spectroscopic analysis of the purified wild-type and mutant PAS domain fragments did not reveal changes in secondary structure content (Figure S7), suggesting that the point mutations do not affect the overall tertiary structure of the molecule. All three single mutations disrupted the mPER2 homodimer under gel filtration conditions (Figure 6A). We therefore propose that the mPER2 homodimer that we observe in the crystal is also formed in solution. The different *K*_D_ values obtained for homodimerization of mPER2[170–473] and the equivalent dPERΔαF fragment cannot be explained based on buried surface areas, which are almost identical for mPER2[170–473] (2,520 Å^2^) and dPERΔαF (2,442 Å^2^, calculated from the dimeric dPER[232–599] structure after deletion of residues 539–599). However, the mPER2 dimer interfaces are characterized by a higher shape complementarity (shape complementarity index of 0.78) [42] than the PAS-A–Trp482 dimer interface of dPER[232–599] (shape complementarity index of 0.69) [31,42]. Moreover, formation of the mPER2[170–473] homodimer is associated with a larger solvation free energy gain (Δ^i^ *G* = −28.3 kcal/mol, including four hydrogen bonds) than homodimer formation of dPERΔαF (Δ^i^ *G* = −21.6 kcal/mol, including ten hydrogen bonds; calculated from the dimeric dPER[232–599] structure after deletion of residues 539–599) [43].

### mPER2 PAS Domain Interactions Are also Formed in Cell Culture

**Figure 7:**
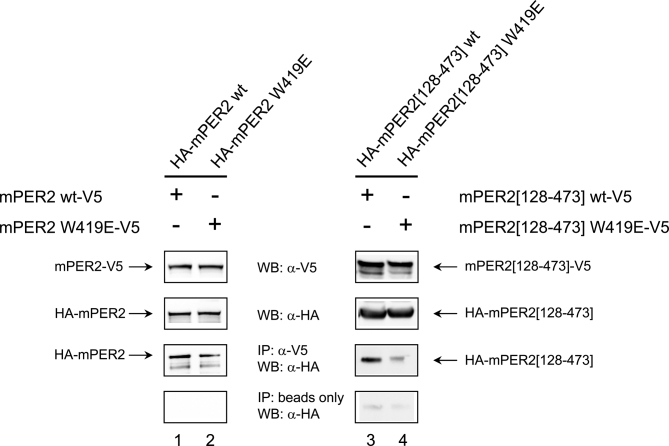
Trp419Glu Mutation Interferes with mPER2 Homodimerization in HEK293 Cells HEK293 cells were transfected with pairs of C-terminally V5- and N-terminally HA-tagged mPER2 full-length proteins or mPER2[128–473] fragments either as wild-type or as W419E variants (lane 1, mPER2 wt-V5/HA-mPER2 wt; lane 2, mPER2 W419E-V5/HA-mPER2 W419E; lane 3, mPER2[128–473]wt-V5/HA-mPER2[128–473]wt; lane 4, mPER2[128–473] W419E-V5/HA-mPER2[128–473]W419E). 48 h after transfection cells were lysed in Co-IP buffer. In the upper two panels of the figure the expression of the corresponding pairs of V5- and HA-tagged mPER2-variants was confirmed by SDS-PAGE/Western blotting using anti-V5- and anti-HA-antibodies, respectively. For immunoprecipitation each cell extract was incubated with anti-V5–antibody and G protein-coupled agarose beads. Co-IP of HA-tagged proteins was analyzed by Western blotting using an anti-HA–antibody (third panel, IP:α-V5, WB:α-HA). In the bottom panels the minus anti-V5-antibody control samples are shown. Blot regions at the migration distance of either full-length mPER2 (lanes 1, 2) or the mPER2[128–473] fragment (lanes 3, 4) are depicted. No unspecific binding of PER2 full-length and PER2 fragment proteins to the beads was detected. The weak band observed in the minus anti-V5–antibody control sample of the mPER2 fragment (lanes 3, 4) is not mPER2, as it is also observed in untransfected HEK293-lysates (not shown).

To establish the role of the tryptophane dimer interaction in cell culture, we have performed Co-IP experiments in HEK293 cells using wild-type and W419E mutant versions of the mPER2[128–473] PAS domain fragment and the full-length mPER2 protein. For both wild-type constructs, mPER2-mPER2 interactions were observed in HEK293 cells, suggesting the mPER2[128–473] fragment to be sufficient for mPER2 homodimer formation in vivo (Figure 7, lanes 1 and 3). The W419E mutation significantly weakened the mPER2 homodimer in the context of the PAS domain fragment (Figures 7, lane 4, and S8, lane 4). We conclude that Trp419 is also involved in mPER2 homodimerization via the PAS domains in HEK293 cells. Moreover, some of our immunoprecipitation experiments indicate, that the W419E mutation also tends to weaken homodimer formation of the full-length mPER2 protein (Figure S8, lane 2). The less pronounced effect of this mutation on full-length mPER2 suggests that in the cell the homodimer might be additionally stabilized via non-PAS mPER2 regions, either through direct mPER2-mPER2 interactions or indirectly by other interacting molecules.

### dTIM Binds to the PAS-B β-sheet Surface of dPER

**Figure 8:**
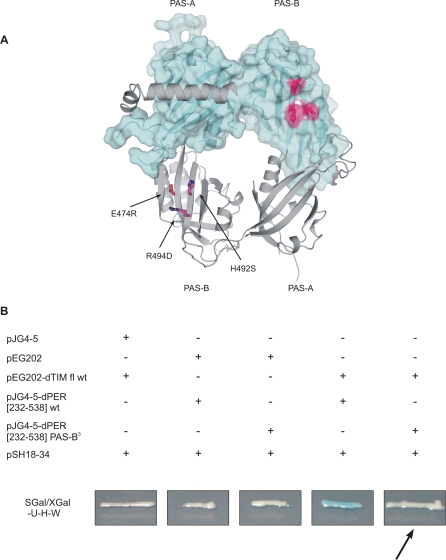
Yeast-Two-Hybrid Analysis of dPER-dTIM Interaction (A) dPER[232–599] structure with residues mutated within the yeast-two-hybrid study highlighted. (B) Yeast-two-hybrid analysis of the interaction between wild-type or mutant versions of dPERΔαF[232–538] and full-length dTIM. Wild-type dPER[232–538] and wild-type dTIM show a strong interaction (see column 4) that is abolished by the introduction of the triple mutation E474R/H492S/R494D (B^3^) in the PAS-B domain (see column 5 and arrow).

Since the PAS-B β-sheet surface is crucial for mPER2 homodimerization, but is uncovered in our dPER[232–599] [31] structure, we reasoned that this surface might be involved in dTIM binding. In support of this assumption, residues 448–512 of dPER corresponding to β-strands βC′, βD′, and βE′ of PAS-B are part of a cytoplasmic localization domain (CLD), which is reported to regulate nuclear entry of the dPER-dTIM complex and is involved in dTIM interactions [12,27]. To test our model for dPER-dTIM interactions, we have introduced mutations E474R, H492S, and R494D in the PAS-B β-sheet surface of dPERΔαF[232–538] and tested the interaction of the mutated dPER fragment with full-length dTIM in yeast-two-hybrid studies (Figure 8). As expected, the PAS-B triple mutation disrupted the dPER-dTIM heterodimer, documenting the importance of the PAS-B β-sheet surface for dTIM binding. Notably, the E474R/H492S/R494D triple mutation did not disrupt the dPER[232–599] homodimer under gel filtration conditions (unpublished data), showing that dPER (unlike mPER2) does not homodimerize via the PAS-B β-sheet surface in solution.

## Discussion

We have undertaken a detailed structural and biochemical analysis of the homo- and heterodimeric PAS domain interactions in *Drosophila* PERIOD (dPER) and its mouse PERIOD homologue mPER2. The mouse PERIOD proteins mPER1, 2, and 3 are known to form homo- and heterodimers in the circadian clock and these interactions are likely mediated via their PAS domains [23,25,26,28]. The PAS domains of dPER mediate interactions with dTIM in the *Drosophila* circadian clock [12,27]. Furthermore, dPER has been shown to form homodimers via its PAS domains in yeast-two-hybrid experiments [32,33], in crystals, and in solution [31]. In a previous x-ray crystallographic study of the dPER[232–599] PAS domain fragment [31], we have shown, that dPER homodimerization is mediated by interactions of the PAS-A β-sheet surface with helix αF in the conserved C-domain as well as a second interface formed between Trp482 of the PAS-B domain and the hydrophobic binding pocket of the PAS-A domain. Using structure-based mutations designed to disrupt the PAS-A–αF interface (mutants M560D, R345E), the PAS-A–Trp482 interface (mutant W482E) or both interfaces (mutant W482E/M560D), the functional significance of the dPER homodimer in the *Drosophila* circadian clock is analyzed in the accompanying report by Landskron et al. [39]. We could show by analytical ultracentrifugation, that the homodimer interaction of dPER is lowered significantly by a factor of about 1,000, when the αF helix is removed; for dimerization of dPER[232–599] we obtained a *K*_D_ of 0.2 μM, for dPERΔαF a *K*_D_ of 256 μM (Figure 3). The importance of the PAS-A–αF interaction in the *Drosophila* clock is clearly demonstrated by the temperature-dependent 29-h phenotype of the *per* ^L^ (V243D) mutation [6] as well as the M560D mutation, which disrupts dPER homodimers in vivo and affects behavioural rhythmicity, nuclear translocation of dPER, and transcriptional repression (see accompanying report by Landskron et al. [39]). In contrast to the strong effect on the homodimerization of full-length dPER in vivo, the M560D mutation only lowered the dimer affinity to about 7 μM when analysed in the context of the purified dPER[232–599] PAS domain fragment (Figure 3). Possibly, the more pronounced effect in vivo is due to additional conformational changes imposed on other dPER regions after weakening of the PAS-A–αF interactions. Since the M560D mutant is still able to bind to dTIM (see accompanying report by Landskron et al. [39]) it is also possible that dTIM binding facilitates monomerization of the mutant in vivo. It is conceivable that dTIM binding to the PAS-B β-sheet surface of dPER (as shown by our yeast-two-hybrid experiments, Figure 8) sterically interferes with binding of the αF helix to PAS-A, which is expected to place dPER regions C-terminal to αF in the direction of PAS-B and hence dTIM. Accordingly, dTIM binding to the PAS-B β-sheet surface would likely lead to a displacement of the αF helix from the PAS-A β-sheet surface (as suggested to be possible by the two αF conformations observed in the dPER[232–599] structure [31]; Figure 2A) and consequently destabilize the dPER homodimer. Weakening of the PAS-A–αF interaction by the M560D mutation may therefore (apart from destabilizing the dPER homodimer) facilitate dTIM binding, which would then further destabilize the dPER homodimer. In this context it is worth mentioning, that the short mutable region, in which several short-period mutations (e.g., *per* ^S^, S589N, 19-h period or *per* ^T^, G593D, 16-h period) [44] and a number of phosphorylation sites [16] have been identified, is located directly C-terminal to the αF helix. Interestingly, phosphorylation of Ser589, i.e., the *per* ^S^ mutation site, has been suggested to inhibit the DBT-dependent phosphorylation of serine residues 607, 613, and 629 as well as threonine 613, resulting in a reduced transcriptional repressor activity but enhanced stability of dPER [45]. It is likely, that phosphorylation of these residues located within 60 amino acids C-terminal to the αF helix is sensitive to whether or not αF is bound to PAS-A. Hence, the phosphorylation status of this region is likely to differ between dPER homodimers (αF bound to PAS-A), dPER-dTIM heterodimers (αF likely displaced from PAS-A), and the M560D mutant (weakened PAS-A–αF interaction). Notably, in the accompanying report by Landskron et al. [39] the M560D mutant protein has been shown to be less phosphorylated and to exhibit a reduced transcriptional repressor activity compared to wild-type dPER. To quantify the contribution of the PAS-A–Trp482 interface to dPER dimerization in solution, we have also analysed the W482E mutant as well as the M560D/W482E double mutant (both in dPER[232–599]) by analytical gel filtration and analytical ultracentrifugation. Whereas the individual W482E mutation resulted in a reduced dimer affinity of about 4 μM, the double mutant completely dissociated the dimer, suggesting that both, the PAS-A–αF- and the PAS-A–Trp482 interface are involved in dimerization in solution. Interestingly, the W482E mutation, which yielded a stable and (according to CD spectroscopy, Figure S1) properly folded protein with a reduced dimer affinity in the context of the dPER[232–599] fragment, lead to an unstable protein in *Drosophila* flies (see accompanying report by Landskron et al. [39]). Likewise, the M560D/W482E double mutant, which lead to a stable and properly folded monomer in the context of the dPER[232–599] fragment (Figure S1) was unstable in *Drosophila* flies. This indicates, that neither the mutations nor monomerization per se are the cause of the reduced stability of the dPER mutants observed in vivo. Rather, other effects, such as the disruption of dTIM interactions in addition to the disruption of the dPER homodimer or changes in the phosphorylation stage, which may ultimately affect binding of the F-box protein SLIMB to dPER [16], are likely to be responsible for the reduced stability observed in vivo. In support of this argumentation, the M560D mutant protein, which is largely monomeric in flies, but still able to interact with dTIM, is stable in vivo. In agreement with our assumption, that in the clock the homodimer stabilizes dPER in absence of dTIM, the PAS-A domain of the monomeric dPERΔαF structure is rather flexible, suggesting that dPER homodimer formation stabilizes the PAS-A domain by insertion of Trp482 into its binding pocket and possibly also by covering the PAS-A β-sheet surface with the αF helix. In the mPER2 structure the PAS-A domain is partially disordered, likely also as a consequence of the missing tryptophane in its binding pocket. However, it has been shown recently, that mPER2 binds heme in the PAS-A domain [46]. Interestingly, Cys215, which has been identified as a heme ligand, is disordered in our mPER2 structure, suggesting that heme binding may induce ordering of this PAS-A domain region. Note however, that the crystallized dPERΔαF- and heme-free mPER2 fragments are properly folded in solution as documented by CD spectroscopy (Figures S1 and S7).

The structure of the PAS domain region of mPER2 revealed a homodimer, which, different from dPER, is mainly stabilized by interactions of the PAS-B β-sheet surface including Trp419 (equivalent to Trp482 in dPER) as a crucial interface residue (Figure 4A and 4B). Interestingly, dPER uses the PAS-B β-sheet surface for dTIM interactions, as demonstrated by our yeast-two-hybrid studies, where the E474R/H492S/R494D triple mutation on this surface disrupts dPER-dTIM interactions (Figure 8). However, the E474R/H492S/R494D triple mutant does not disrupt the dPER[232–599] homodimer under gel filtration conditions (unpublished data), showing that dPER does not homodimerize via the PAS-B β-sheet surface in solution. In contrast, the single mutations of Trp419, Ile427, or Phe415 in the PAS-B dimer interface of mPER2 to Glu disrupted mPER2 homodimers under gel filtration conditions (Figure 6A). Furthermore, the W419E mutation disrupted homodimers of an mPER2 PAS domain fragment in HEK293 cells (Figure 7). We therefore believe that mPER2 also uses the PAS-B β-sheet surface for homodimer formation in solution and inside living cells. Formation of a dPER-like homodimer does not seem likely for the mPER proteins, since the hydrophobic pocket of PAS-A, which embeds Trp482 in dPER, is somewhat more closed in mPER2. Apart from movements of the βA- and βB-strands, Ala287 is replaced by a bulkier histidine (His238) in mPER2, which is likely to interfere with Trp binding in the hydrophobic pocket of PAS-A (Figure 5A). Whereas Ala287 is conserved as an alanine in insect PER proteins (e.g., from the monarch butterfly [Q7Z0C9], the silk moths Bombyx mori [Q1A177] or Antheraea pernyi [Q17062], the honeybee [Q9NDF3], the red flour beetle [A9XCF2], the house fly [Q9UA11], and *Drosophila* flies [P07663]), it is replaced by a bulky tyrosine in mPER1 and mPER3 (Figure 1B) as well as other vertebrate PER proteins such as Zebrafish (z) and *Xenopus* (x) PER proteins (zPER1/Q7sZZ4; zPER2/Q7T1C9; zPER3/B3DK47; zPER4/B3DK47; xPER1/Q9DFU8; xPER2/Q9DG29; xPER3/Q08CY0). Furthermore, residues in the PAS-B dimer interface are generally conserved between the three mouse PERIOD homologues as well as other vertebrate PER proteins. Finally, as part of the conserved C-domain, the αF helix of dPER is conserved in insect PER proteins but not in vertebrate PER proteins. Conversely, the region N-terminal to the PAS-A domain corresponding to residues 170–189 of mPER2 is well conserved between vertebrate PER proteins, but not in insect PER proteins. On the basis of this sequence comparison, we propose that the dPER structure including the PAS-A–αF- and the PAS-A–Trp482 homodimer interface [31] provides a model for insect PER proteins, whereas homo- and heterodimers formed by mammalian and possibly other vertebrate PERIOD proteins involve the PAS-B dimer interface revealed by our mPER2 structure.

In the mammalian clock, mPER-mPER heterodimer formation has been implicated in the regulation of cellular shuttling of mPER proteins, with different results obtained in different cell culture systems. For example in COS7 cells, mPER3 was found to be responsible for nuclear import of mPER1 and mPER2 following direct serum shock induced interactions via the PAS domains [25]. The same study shows that mPER2 and mPER3, but not mPER1, form homodimers with and without serum shock. Interestingly, the PAS-B β-sheet surface (β-strands C′, D′, and E′) is reported to contain a functionally active CLD in mPER3 [25], which may be masked upon interaction with mPER1 or mPER2. This situation is reminiscent of earlier reports on dPER, according to which a CLD (also located in β-strands C′, D′, and E′) is covered by dTIM upon heterodimerization, promoting nuclear import of the dPER-dTIM complex [12]. While our yeast-two-hybrid studies provide clear evidence for dTIM binding to the PAS-B β-sheet surface of dPER including the CLD region, our mPER2 structure suggests how mPER1/2-mPER3 heterodimerization could mask the CLD of mPER3 through antiparallel interactions of the PAS-B β-sheet surfaces.

Different from mPER3, the CLD region of mPER1 (strands βC′, βD′, βE′ of PAS-B) is not functional, i.e., does not contribute to the cytoplasmic retention of mPER1 in HEK293 cells. Instead, a functional NES has been identified in mPER1 and mPER2 [40] in a region that corresponds to the αE helix in our mPER2 structure. A subsequent study [20] showed, that the CLD of mPER2 is also not functional in COS7 cells and identifies two additional functional NES sequences (apart from the NES in the αE helix) in the N- and C-terminal region of mPER2. Within the NES sequence in the αE helix of our mPER2 structure, NES residues Leu460, Ile464, and Leu467 pack against the αC′ helix of PAS-B, whereas Met469 points to the molecule surface and is involved in homodimer interactions (Figures 4D and 5B). These features are typical for NES sequences located in amphipathic α-helices, with the first three residues (Leu460, Ile464, Leu467) being buried and the last residue (Met469) pointing to the outside for exportin interactions [41]. On the basis of our structural comparison with dPER we propose that an equivalent NES function could be carried out by αE residues Ile526, Ile530, and Leu534 of dPER (corresponding to Leu460, Ile464, and Leu467 of mPER2) as well as Val538_dPER_ functionally replacing Met469_mPER2_ (Figure 5C). This NES motif might play a role in the reported Crm1-dependent nuclear export of dPER [34]. Although the importance of Met469 for nuclear export of mPER2 has been clearly demonstrated by its mutation to lysine [40], our structural comparison with dPER (Figure 5C) suggests that Val472_mPER2_ equivalent to Val538_dPER_ might also be involved in interactions with the export machinery. In mPER1, the I493A/L496A/L498K triple mutation in the αE region had the same destructive effect on nuclear export activity as the corresponding triple mutation I464A/L467A/M469K analysed in mPER2 [40]. In contrast, the single mutation of Leu498_mPER1_ (corresponding to Met469_mPer2_) to Lys had no effect on nuclear export activity [40]. On the basis of our PERIOD structures and sequence alignment we therefore suggest that Val502_mPER1_ equivalent to Val538_dPER_ might play a role in exportin interactions of mPER1. It is conceivable that homo- or heterodimerization of the mPER proteins in the way we see it in our mPER2 crystal structure would interfere with binding to the export complex, providing a possible explanation for enhanced nuclear import of some mPER complexes [25,26]. Our mPER2 structure therefore suggests how the CLD region and the NES in the αE helix of mPER proteins can be masked by the formation of homo- or heterodimeric mPER complexes and thereby adds to the understanding of mammalian clock regulation via cellular shuttling of clock proteins.

For the bHLH-PAS transcription factors aryl hydrocarbon receptor nuclear translocator (ARNT) and its heterodimerization partner hypoxia inducible factor 2α (HIF-2α) it has been shown, that homodimerization of ARNT as well as HIF-α-ARNT heterodimerization occurs via the PAS-B β-sheet surfaces, which pack against each other in an antiparallel manner, similar to mPER2 [47,48]. Interestingly, ARNT has a tyrosine and HIF-α an arginine instead of Trp419_mPER2._ However, in the ARNT-ARNT homodimers as well as the ARNT-HIF-2α heterodimers, the PAS-B domains interact via a much larger surface area (2,378 Å^2^ of buried surface area for ARNT-HIF-2α; 1,742 Å^2^ for ARNT-ARNT) than in mPER2 (1,217 Å^2^ for the PAS-B domains only), placing the Trp419_mPER2_ equivalent Tyr and Arg residues at the edge of the main dimer interface. For homodimerization of the ARNT PAS-B domains, a *K*_D_ of about 500 μM was obtained by nuclear magnetic resonance (NMR) titration experiments, for the HIF-2α-ARNT PAS-B heterodimer, the *K*_D_ was 30 μM [48]. In comparison, the *K*_D_ that we obtained for our mPER2 fragments including both the PAS-A and the PAS-B domain is about 2 μM. We conclude that in mPER2 the PAS-A domain significantly increases the homodimer affinity despite the less tight PAS-A dimer interactions observed in our crystal structure. This conclusion is supported by the fact, that the solvation free energy gain for mPER2[170–473] homodimer formation (Δ^i^ *G* = −28.3 kcal/mol, including four hydrogen bonds) is significantly larger than for the isolated PAS-B domain of mPER2 (Δ^i^ *G* = −14.5 kcal/mol, no hydrogen bonds assigned), the HIF-2α–ARNT PAS-B heterodimer (Δ^i^ *G* = −21.1 kcal/mol, including 21 hydrogen bonds and 17 salt bridges) and the ARNT-ARNT PAS-B homodimer (Δ^i^ *G* = −10.6 kcal/mol, including eight hydrogen bonds and eight salt bridges) [43].

Interestingly, a recent study has shown, that the Trp482_dPER_ equivalent tryptophane residue (Trp362_mCLOCK_) is crucial for the function of the bHLH-PAS transcription factor mCLOCK [49]. Moreover, mutations G332E, H360Y, Q361P, W362R, and E367K in the PAS-B β-sheet surface of mCLOCK interfere with binding of mCRY, but not of mBMAL1 to mCLOCK [16,49]. The different modes of homo- and heterodimer formation observed for dPER [31], mPER2 (this study), as well as HIF-2α and ARNT [47,50] raise the question, whether mCLOCK-mBMAL1 heterodimers might be formed in one of the described or yet another way. The mutational analysis of mCLOCK clearly shows that mCLOCK uses the PAS-B domain not only (if at all) for PAS-PAS domain interactions with mBMAL1, but also for heterotypic interactions with mCRY. Future structural, biochemical, and biophysical analyses of mPER proteins and bHLH-PAS transcription factors will help to understand the differences and similarities between the tandem PAS-repeat regions of these clock components.

In contrast to dPER, mPER2 does not require the region corresponding to the αF helix for homodimer formation in solution, as evidenced by the fact that the mPER2[128–473] and mPER2[170–473] PAS domain fragments lacking this region dimerize with 2.25 and 1.34 μM affinities, respectively, and behave as homodimers in gel filtration experiments (Figure 6). Interestingly, the αF equivalent region of mPER2 contains a phosphodegron (^477^SpSGYGpS^482^), which has been shown to mediate phosphorylation-dependent binding of the ubiquitin ligase adaptor protein β-TrCP (β-transducin repeat-containing protein) [51,52] and therefore proteasomal degradation of mPER2. In good agreement with this changed role of the αF equivalent region in mPER2, the intermolecular PAS-A-αF interaction observed in the dPER structure is replaced by an intramolecular interaction of the PAS-A β-sheet surface of mPER2 with residues located N-terminal to the PAS-A domain (Figures 4A, S5, and S6). This N-terminal capping of the PAS domain has also been observed in the photoactive yellow protein (PYP) [53] as well as the circadian clock protein Vivid (Figure S6) [54]. While in these proteins the N-terminal cap is implicated in light signalling, the N-terminal cap region in mPER2 mainly seems to stabilize the protein as suggested by the fact that a fragment starting directly at the PAS-A domain is insoluble (unpublished data).

N-terminal residues 128–169 do not contribute to homodimerization of mPER2 as shown by the comparable *K*_D_ values of 2.25 μM for mPER2[128–473] and 1.34 μM for the crystallized mPER2[170–473] fragment. However, since the W419E mutation disrupted mPER2[128–473] homodimers much more efficiently than full-length mPER2 homodimers in our Co-IP experiments in HEK293 cells (Figure 7), the dimer appears to be additionally stabilized via non-PAS–mPER2 regions in the cellular context, either through direct mPER2-mPER2 homodimer interactions or indirectly via other interacting proteins.

It has been reported previously, that the clock proteins mCRY1 and mCRY2 bind to the most C-terminal region of mPER2 [20,55,56] and that the kinase CKIɛ binds to an mPER2 region comprising residues 554–763 C-terminal to the PAS domains [57]. These proteins might therefore be possible candidates for an indirect stabilization of the mPER2 homodimer. Indeed, huge (>1 MDa) mPER1 and mPER2 containing complexes have been observed in mouse liver nuclear extracts [58], in which mCRY1 and 2, the RNA- and DNA-binding protein NONO and the histone methyltransferase subunit WDR5 were also detected.

Although many more protein-protein interactions are important for mammalian and *Drosophila* circadian clock function, we have, by structurally and biochemically describing the homo- and heterodimeric PAS domain interactions of dPER and mPER2, taken an important step towards a quantitative understanding of the protein interaction networks in these circadian oscillators.

## Materials and Methods

### Protein preparation and crystallization.

All *Drosophila* PERIOD (dPER) and mouse PERIOD2 (mPER2) fragments were overexpressed as recombinant GST-fusions in the Escherichia coli strains BL21(DE3) or Rosetta(DE3) using a pGEX-6P2 expression vector with a PreScission protease recognition site for removal of the GST-tag. The proteins were purified via GSH affinity and gel filtration columns. For crystallization, gel filtration, and analytical ultracentrifugation, the GST-tag was cleaved and quantitatively removed.

### Crystallization.

Crystals of dPER[232–538] diffracting to 4.0-Å resolution were grown at 4 °C in hanging drop setups using a reservoir solution with 70 mM sodiumtartrate and a protein solution with 10 mg/ml dPER[232–538], 20 mM HEPES (pH 7.7), and 5 mM DTE. The dPER[232–538] crystals belong to space group P3(2)21 with unit cell constants *a* = *b* = 114.95 Å, *c* = 85.7 Å, and one molecule per asymmetric unit (solvent content 70%). For data collection at 100 K, crystals were transferred into a cryoprotecting solution containing 20% ethyleneglycol, 20 mM HEPES (pH 7.7), 5 mM DTE, and shock frozen in liquid nitrogen.

Crystals of mPER2[170–473] diffracting to 2.4-Å resolution were grown at 20 °C in hanging drop setups using a reservoir solution with 23% PEG8000 and 100 mM HEPES (pH 7.7) and a protein solution with 10 mg/ml mPER2[170–473], 25 mM HEPES (pH 7.7), 200 mM NaCl, and 2 mM DTE. The crystals belong to space group P2_1_ with unit cell constants *a* = 69.67 Å, *b* = 63.38 Å, *c* = 72.51 Å, β = 102.25 °, and two molecules per asymmetric unit (solvent content 44.9%). For data collection at 100 K, crystals were transferred into a cryoprotecting solution containing 30% PEG400 (v/v) and shock frozen in liquid nitrogen.

### Data collection, structure determination, and refinement.

A 4.0-Å dataset of a single dPER[232–538] crystal was collected at beamline ID29 (ESRF) and a 2.4-Å dataset of a single mPER2[170–473] crystal at beamline X10SA (SLS). All datasets were processed with XDS [59]. The structure of dPER[232–538] was solved by molecular replacement using AMORE [60] with one monomer of the refined dPER[232–599] structure with helix αF deleted as search model. The mPER2[170–473] structure was solved by molecular replacement using MOLREP [61]. The search model was one monomer of the dPER[232–599] structure with nonconserved residues mutated to alanine. Helix αE and the preceding linker as well as nonconserved loop regions and helix αF were deleted from the model. The structures were refined with CNS [62] applying several rounds of simulated annealing, positional, and B-factor refinement followed by model building into 2*F*_o_ − *F*_c_ and *F*_o_ − *F*_c_ maps using the programs XFit [63], O [64], or Coot [65]. Simulated annealing omit maps were regularly calculated in CNS and used to verify, correct, or build the model.

The final dPER[232–538] model consists of 257 amino acids. Residues 232–237, 295–308, 321–333, and 347–363 are not seen in the electron density because of conformational disorder. The Ramachandran plot depicts 98.7% of the main chain torsion angles in the most favoured and allowed regions, with three residues in disallowed regions.

The final mPER2[170–473] model consists of 519 amino acids and 198 water molecules. Residues 183–186, 214–220, 251–264, 277–280, and 297–304 of molecule 1 and residues 170–178, 186–187, 213–221, 248–262, 276–282, 296–302, and 450–454 of molecule 2 are not seen in the electron density owing to conformational disorder. The N terminus of molecule 1 contains two additional residues (Gly168 and Ser169), which are cloning artefacts. Residues 182 and 276 of molecule 1 and residues 181–185, 204, 212, 263, 283, 295, 303, and 449 of molecule 2 are modelled as Ala due to conformational disorder of their side chains. The Ramachandran plot depicts 100% of the main chain torsion angles in the most favoured and allowed regions with 0 residues located in disallowed regions. All structures exhibit good stereochemistry (Table 1).

**Table 1:**
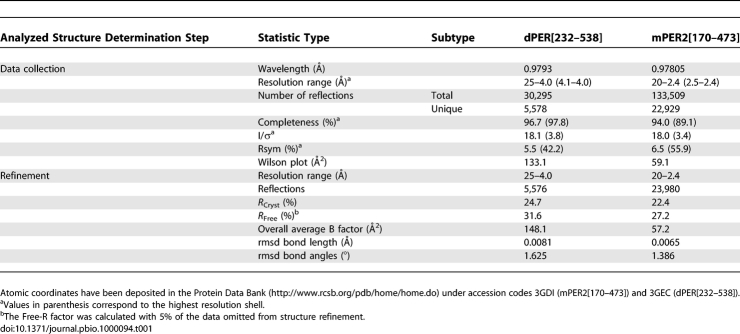
X-Ray Data Collection and Refinement Statistics

Figures were generated with Pymol (http://www.pymol.org). Superpositions were carried out with the combinatorial extension (CE) method [66]. Buried surface areas and solvation free energy (Δ^i^ *G*) calculations were carried out with the PISA server (Protein interfaces, surfaces, and assemblies service PISA at European Bioinformatics Institute, http://www.ebi.ac.uk/msd-srv/prot_int/pistart.html) [43]. Δ^i^ *G* includes the solvation free energy owing to hydrophobic interactions (as provided by the PISA server) as well as hydrogen bonds and salt bridges across the interface assuming a contribution of 0.5 kcal/mol for each hydrogen bond and 0.3 kcal/mol for each salt bridge.

### Site directed mutagenesis.

Mutants were constructed using the Quickchange site-directed mutagenesis kit (Stratagene) and sequenced. For analytical gel filtration and ultracentrifugation, the mutant constructs were transformed into E. coli BL21(DE3) or Rosetta(DE3) cells, expressed to high levels, and purified to homogeneity essentially as described for the wild-type protein.

### Analytical gel filtration chromatography.

Analytical gel filtration chromatography of purified GST-free dPER and mPER2 fragments was carried out at 4 °C on an ÄKTA Explorer system using a HiLoad 16/60 Superdex 200 prep grade column or a 10/30 Superdex 75 column (Amersham Biosciences) preequilibrated with a buffer containing 25 mM HEPES (pH 7.7), 200 mM NaCl, and 2 mM DTE. Protein samples of 1 ml containing 5–10 mg/ml protein (16/60) or 100 μl containing 350–450 μg protein (10/30) were loaded on the column. Elution from the column was monitored by measuring absorbance at 280 nm. A calibration curve was generated by measuring the elution volumes of a series of standard proteins of known molecular mass. Molecular masses of dPER and mPER2 proteins were estimated by interpolating their elution volumes onto the calibration curve and comparing the elution volumes of the fragments relative to each other.

### Analytical ultracentrifugation.

Experiments were conducted in an Xl-I analytical ultracentrifuge (BeckmanCoulter), using the interference optics of the instrument. Concentrations of the stock solutions ranged from 0.8–2 mg/ml. All experiments were performed using a buffer containing 25 mM HEPES (pH 7.7), 100 mM NaCl, and 2 mM DTE. Buffer in dialysis equilibrium, achieved via gel filtration chromatography, was used for dilution and optical referencing. Auxiliary parameters were calculated from composition using SEDNTERP as described [31,67]. A conversion factor of 3.29 fringes/mg/ml was used to convert signal units to mass and molar concentrations, using the calculated masses of the respective proteins. All software used can be freely downloaded from http://www.rasmb.bbri.org/rasmb/ and http://www.biotech.uconn.edu/auf/.

Sedimentation equilibrium (SE) experiments were performed in the following way: Four different starting concentrations were prepared by using the stock solution undiluted, 2-, 5-, and 10-fold diluted. Depending on starting concentrations, undiluted/2-/4-/8-fold dilutions were also prepared. 120–150 μl were brought to apparent chemical and sedimentation equilibrium, as judged by the software MATCH, at three or four speeds ranging from 10,000–30,000 rpm, depending on the molar masses of the system. All experiments were performed at 10 °C. Data were fitted to various models using NONLIN. The best model was selected on the basis of a minimal rmsd compared to all other models evaluated and by randomness of the residuals. Various starting guesses for the fitted parameters were used to test their robustness. Values reported are independent of starting guesses within the given confidence limits.

### CD Spectroscopy.

A purified protein sample was diluted to a final concentration of 5 μM in 20 mM NaH_2_PO_4_ (pH 7.7), 20 mM Na_2_SO_4_ buffer. CD spectra were measured by a Jasco J-815 spectropolarimeter and represent the mean molar ellipticity per amino acid residue of protein after buffer correction. Data were collected at 10 °C in a range from 190 nm to 260 nm in 0.5-nm intervals collecting data for 0.5 s at each point. For each measurement ten spectra were used for accumulation.

### Yeast-two-hybrid.

Yeast-two-hybrid experiments were carried out as described [68]. All dPER fragments were subcloned as prey into the pJG4–5 plasmid (http://www.invitrogen.com). Full-length dTIM was subcloned as bait into the pEG202 plasmid [69]. Cotransformation of two-hybrid vectors and a lacZ-encoding helper plasmid (http://www.invitrogen.com) into the yeast strain EGY48 (http://www.invitrogen.com) was performed as described previously [70]. Transformants were plated on synthetic dextrose (SD) medium lacking uracil, histidine, and tryptophane. To assay for the lacZ reporter, transformants were grown on galactosidase and X-Gal–containing medium lacking uracil, histidine, and tryptophane. Plates were incubated for 2–3 d at 30 °C. Same results were obtained in three different experiments under the same conditions.

### Cell culture and transfection.

HEK293 cells were maintained in DMEM supplemented with 10% fetal bovine serum and 100 μg/ml penicillin and streptomycin. Transfection was carried out with the CalPhos Mammalian Transfection kit (Clontech Laboratories) according to the manufacturer's protocol. HEK293 cells were plated in 75-cm^2^ flasks. After reaching of 80%–90% confluency cells were provided with 12 ml of fresh medium and transfection was performed. A total of 36 μg of DNA (composed of 18 μg V5-tagged and 18 μg HA-tagged PER2 variant constructs) was transfected per flask.

### Co-IP.

Cells were harvested 24–48 h after transfection in iced Co-IP buffer (20 mM Tris-HCl [pH 8], 140 mM NaCl, 1.5 mM MgCl_2_, 1 mM TCEP, 1% Triton X-100, 10% glycerine). To remove cell debris the lysates were centrifuged for 30 min at 13,000 rpm and 4 °C. Protein amount was determined using the BCA method. For the immunoprecipitation 1 mg protein in a volume of 500 μl was incubated with anti-V5 antibody (Invitrogen) and G protein coupled agarose beads (Santa Cruz Biotechnology). To control for unspecific binding of mPER2 full-length or fragment proteins to the beads identical samples without adding anti-V5 antibody were processed in parallel. Incubation was performed for at least 18 h at 4 °C in a rotator. Subsequently, the beads were washed three times with 500 μl of iced Co-IP washing buffer (20 mM Tris-HCl [pH 8], 150 mM NaCl, 0.5% IGEPAL CAG-30). Immunoprecipitates were eluted from the beads by adding 30 μl SDS-PAGE sample buffer and heating for 10 min at 95 °C. 5 μl of the eluates were analyzed by Western blotting.

### Western blotting.

For the input control equal amounts of protein and after Co-IP 5 μl of the eluates were loaded on 8% or 12% polyacrylamide gels and separated by SDS-PAGE. After transferring the proteins to nitrocellulose membranes detection of V5-tagged proteins and HA-tagged proteins was carried out using mouse anti-V5 antibody (1:5,000, Invitrogen) and mouse anti-HA antibody (1:1,000, Roche), respectively. Signal intensity was determined with the Lumi-Imager (Roche). Blotting performance was monitored by Ponceau staining.

## Supporting Information

Figure S1 CD Spectra of Wild-Type and Mutant dPER PAS Domain Fragments: Dimer Interface Mutations Do Not Affect the Overall Structure of the Molecule (221 KB TIF)

Figure S2 Stereo View of the mPER2[170–473] Homodimer Structure The two orientations presented in (A) and (B) are related by a 90 ° rotation. Trp419 is highlighted as atomic stick figure.
(1.69 MB TIF)

Figure S3 Close-Up Views of the PAS-B β-sheet Dimer Interface (A) Close-up view of the dimer interactions of Trp419 with the PAS-B β-sheet surface (same orientations as in Figure 4B [left] and 4C [right]). Interacting residues Trp419, Pro418, as well as Pro390, Ser411, Ser413, Ile427, and Arg429 are highlighted as atomic stick figure. Whereas Ser411 of molecule 1 forms a direct hydrogen bond to Trp419 of the dimerizing molecule (left), Ser411 of molecule 2 interacts with Trp419 via a water molecule (right). Left, molecule 1 in dark blue, molecule 2 in grey; right, molecule 2 in dark blue, molecule 1 in grey. The 1 sigma 2fo-fc composite omit map is shown in blue, water molecules as red spheres.
(B) Electrostatic surface representation of the PAS-B dimer interface of mPER2 highlighting the hydrophobic nature of the interface. One molecule is shown as ribbon presentation with interface residues Trp419, Phe415, and Phe425 as atomic stick figure.
Found at doi:10.1371/journal.pbio.1000094.sg003 1.92 MB TIF).

Figure S4 Superposition of Molecule 1 (Dark Blue) and Molecule 2 (Grey) of mPER2[170–473] The two orientations are related by a 180 ° rotation.
(891 KB TIF)

Figure S5 Close-Up View of mPER2 Molecule 1 Showing Interactions of the PAS-A Domain (Dark Blue) with the N-Terminal Cap Region (Orange) Interacting residues Tyr204, Val294, and Trp249 of the PAS-A domain (dark blue) as well as residues Tyr171, Val176, and Glu177 in the N-terminal cap region (orange) are shown as atomic stick figures.
(722 KB TIF)

Figure S6 Surface Representation of the mPER2 Dimer (Black and Grey) Showing the N-Terminal Cap Region (Orange, Ribbon Representation) Covering the PAS-A β-sheet Surface Elements of other known structures covering a very similar part of the PAS domain β-sheet surface are superimposed on the PAS-A domain of mPER2: the C-terminal αF helix of dPER (1WA9, aa 543–575) [31], the C-terminal αJ helix of *As*LOV2 (2V0W, aa 527–546) [71,72], the N terminus of *Eh*PYP (1NWZ, aa 1–27) [73], the N terminus of *Nc*Vivid (2PD7, aa 36–73) [54]*,* the N terminus of *Av*NifL (2GJ3, aa 22–36) [74], the N terminus of *Ec*DOS (1S66, aa 16–31) [75], the N terminus of *Np*STHK (2P04, aa 1–19) [76], and the N terminus of *Rm*FixL (1EW0, aa 122–147) [77]*.*
(2.41 MB TIF)

Figure S7 CD Spectra of Wild-Type and Mutant mPER2 PAS Domain Fragments: Dimer Interface Mutations Do Not Affect the Overall Structure of the Molecule (193 KB TIF)

Figure S8 In HEK293 Cells the Trp419Glu Mutation Weakens Homodimerization in mPER2 Fragment and Full-Length Protein HEK293 cells were transfected with C-terminally V5-tagged mPER2 full-length protein or mPER2[128–473] fragment either as wild-type or as W419E variant (lane 1, mPER2 wt-V5; lane 2, mPER2 W419E-V5; lane 3, mPER2[128–473]wt-V5; lane 4, mPER2[128–473]W419E-V5). 48 h after transfection cells were lysed in Co-IP buffer. In the upper panel of the figure the expression of the corresponding V5-tagged mPER2-variants was confirmed by SDS-PAGE/Western blotting using anti-V5–antibody. For Co-IP each cell extract was incubated with anti-V5–antibody and G protein coupled agarose beads. Co-IP of V5-tagged proteins was analyzed by Western blotting using an anti-V5–antibody (see IP, αV5; WB, αV5, middle panel). The Co-IPs using the W419E mutant variants show a less intensive anti-V5–antibody signal because of the fact that lower amounts of homodimer are precipitated on the beads. Residual intensity results from the residual monomers binding to the beads. In the bottom panels the minus anti-V5–antibody control samples are shown. Blot regions corresponding to the migration distances of either full-length mPER2 (lanes 1, 2) or the mPER2[128–473] fragment (lanes 3, 4) are depicted. No unspecific binding of PER2 full-length and PER2 fragment proteins to the beads was detected.
(662 KB TIF)

## Abbreviations

ARNT: aryl hydrocarbon receptor nuclear translocator
CD: circular dichroism
CKIɛ: casein kinase Iɛ
CLD: cytosolic localization domain or cytoplasmic localization domain
Co-IP: co-immunoprecipitation
CRY: CRYPTOCHROME
d: Drosophila
DBT: Doubletime
HIF: hypoxia inducible factor
m: mouse
NES: nuclear export signal
PAS: PER-ARNT-SIM
PER: PERIOD
rmsd: root mean square deviations
TIM: TIMELESS
